# HER2-specific immunoligands engaging NKp30 or NKp80 trigger NK-cell-mediated lysis of tumor cells and enhance antibody-dependent cell-mediated cytotoxicity

**DOI:** 10.18632/oncotarget.5135

**Published:** 2015-09-15

**Authors:** Matthias Peipp, Stefanie Derer, Stefan Lohse, Matthias Staudinger, Katja Klausz, Thomas Valerius, Martin Gramatzki, Christian Kellner

**Affiliations:** ^1^ Division of Stem Cell Transplantation and Immunotherapy, 2^nd^ Department of Medicine, Christian-Albrechts-University of Kiel, Kiel, Germany

**Keywords:** NK cells, NKp30, NKp80, B7-H6, ADCC

## Abstract

NK cells detect tumors through activating surface receptors, which bind self-antigens that are frequently expressed upon malignant transformation. To increase the recognition of tumor cells, the extracellular domains of ligands of the activating NK cell receptors NKp30, NKp80 and DNAM-1 (i.e. B7-H6, AICL and PVR, respectively) were fused to a single-chain fragment variable (scFv) targeting the human epidermal growth factor receptor 2 (HER2), which is displayed by various solid tumors. The resulting immunoligands, designated B7-H6:HER2-scFv, AICL:HER2-scFv, and PVR:HER2-scFv, respectively, bound HER2 and the addressed NK cell receptor. However, whereas B7-H6:HER2-scFv and AICL:HER2-scFv triggered NK cells to kill HER2-positive breast cancer cells at nanomolar concentrations, PVR:HER2-scFv was not efficacious. Moreover, NK cell cytotoxicity was enhanced synergistically when B7-H6:HER2-scFv or AICL:HER2-scFv were applied in combination with another HER2-specific immunoligand engaging the stimulatory receptor NKG2D. In contrast, no improvements were achieved by combining B7-H6:HER2-scFv with AICL:HER2-scFv. Additionally, B7-H6:HER2-scFv and AICL:HER2-scFv enhanced antibody-dependent cell-mediated cytotoxicity (ADCC) by the therapeutic antibodies trastuzumab and cetuximab synergistically, with B7-H6:HER2-scFv exhibiting a higher efficacy. In summary, antibody-derived proteins engaging NKp30 or NKp80 may represent attractive biologics to further enhance anti-tumor NK cell responses and may provide an innovative approach to sensitize tumor cells for antibody-based immunotherapy.

## INTRODUCTION

Natural killer (NK) cells are innate immune cells playing a major role in the host's early defense against infections and tumors [1, 2]. They are attractive targets for immunotherapy of cancer as they are able to recognize and kill malignant cells by natural cytotoxicity and produce a variety of immunoregulatory cytokines, which shape both innate and adaptive immune responses [3, 4]. For discrimination between healthy and stressed cells NK cells express a repertoire of germline-encoded receptors with either stimulatory or inhibitory functions, which in a complex interplay enable NK cells not only to discriminate between healthy and stressed cells, but also to control their effector functions [5, 6]. Thus, NK cells are triggered by contact with stressed cells expressing either reduced amounts of self proteins, which are recognized by inhibitory NK cell receptors (missing self recognition), and/or increased amounts of self proteins engaging stimulatory NK cell receptors (induced self recognition). In addition, NK cells express the low affinity Fcγ receptor for immunoglobulins (FcγRIIIa) and mediate antibody-dependent cell-mediated cytotoxicity (ADCC), which is considered an important effector mechanism of many therapeutic antibodies [4, 7–9].

NK cell surface receptors promoting natural cytotoxicity include natural killer group 2 member D (NKG2D; CD314), NKp30 (CD337), NKp44, (CD336), NKp46 (CD335), NKp80 (killer cell lectin-like receptor subfamily F, member 1), DNAX accessory molecule 1 (DNAM-1; CD226) and others [1, 6]. Their ligands include diverse self-proteins which are expressed upon cellular stress, malignant transformation, viral infections or upon activation, and function as danger signals alerting NK and other effector cells. Among the varying cellular ligands for activating NK cell receptors, the ligands for NKG2D comprising MHC class I related chains A and B as well as UL-16 binding proteins (ULBP) 1–6 are the best characterized [10, 11]. In recent years the ligands for additional NK cell receptors were identified and were shown to be expressed or up-regulated by tumors of different entities and to trigger NK cell mediated killing of malignant cells. Among those are Nectin-2 (CD112) and the poliovirus receptor (PVR, CD155), which both were shown to engage DNAM-1 [12–15], two cellular ligands for NKp30, human leukocyte antigen-B-associated transcript 3 and B7-H6 [16–18], as well as activation-induced C-type lectin (AICL) ligating NKp80 [19, 20].

Despite the linkage between malignant transformation and the expression of alert signals provoking anti-tumoral immune responses, tumor cells are often not efficiently recognized by the immune system. Thus, tumor cells were reported to evade NK cell cytotoxicity by immune shaping or immune suppression. For example, tumors may escape NK cell recognition by down-modulation or shedding of danger signal molecules or by up-regulation of inhibitory HLA molecules [21–25]. Thus, strategies maintaining or restoring NK cell recognition of tumors may represent an innovative treatment option. With the aim to increase the visibility of tumors to NK cells, recombinant tumor cell-directed immunoligands have been developed, which contain a single-chain fragment variable (scFv) as tumor targeting device and a ligand of an activating surface receptor to engage NK cells [26–28]. With the immunoligands binding to cell surface antigens via the scFv the tumor cells are coated with the danger signal molecule resulting in a mimicked ‘induced self’ phenotype, that is required for recognition and elimination of malignant cells by NK cells. Previously, we have shown that CD20 directed immunoligands that were equipped with ULBP2 and B7-H6 and engaged NKG2D and NKp30, respectively, triggered NK cell-mediated killing of lymphoma and leukemia cells and enhanced ADCC by monoclonal antibodies [29–31].

Apart from B7-H6 and NKG2D-specific ligands, ligands for other activating receptors such as NKp80 or DNAM-1 have not been tested yet as functional domains of recombinant immunoligands, and studies with B7-H6 are limited to CD20. The promising results obtained so far motivated us to investigate also HER2 (CD340) as a target antigen on solid tumors [9] and to test immunoligands containing AICL, B7-H6, and PVR as effector domains in this model system. Thus, immunoligands were generated by fusing the extracellular domains of these ligands to a HER2-specific scFv and analyzed for their abilities to trigger NK cell cytotoxicity and to boost ADCC.

## RESULTS

### Generation of immunoligands and specific binding

The extracellular domains of B7-H6, AICL and PVR were genetically fused to a HER2-specific scFv derived from the humanized antibody humAb4D5-8 (Suppl. Fig. 1A). The resulting fusion proteins, designated as B7-H6:HER2-scFv, AICL:HER2-scFv and PVR:HER2-scFv, respectively, were expressed in human cells and purified from cell culture supernatants by affinity chromatography (Suppl. Fig. 1B). Potential remaining contaminants or protein aggregates were removed by gel filtration, resulting in homogenous protein preparations (Suppl. Fig. 1C). The immunoligands were specifically detected by western blotting using specific antibodies recognizing the histidine-tag (Suppl. Fig. 1D), revealing apparent molecular masses of approx. 75–100 kDa, 50–60 kDa and 100–120 kDa for B7-H6:HER2-scFv, AICL:HER2-scFv and PVR:HER2-scFv, respectively. Enzymatic deglycosylation revealed that all fusion proteins were significantly glycosylated (Suppl. Fig. 1D), which had accounted for differences between the experimentally determined and the predicted molecular masses of 58 kDa (B7-H6:HER2-scFv), 45 kDa (AICL:HER2-scFv) and 65 kDa (PVR:HER2-scFv).

**Figure 1:**
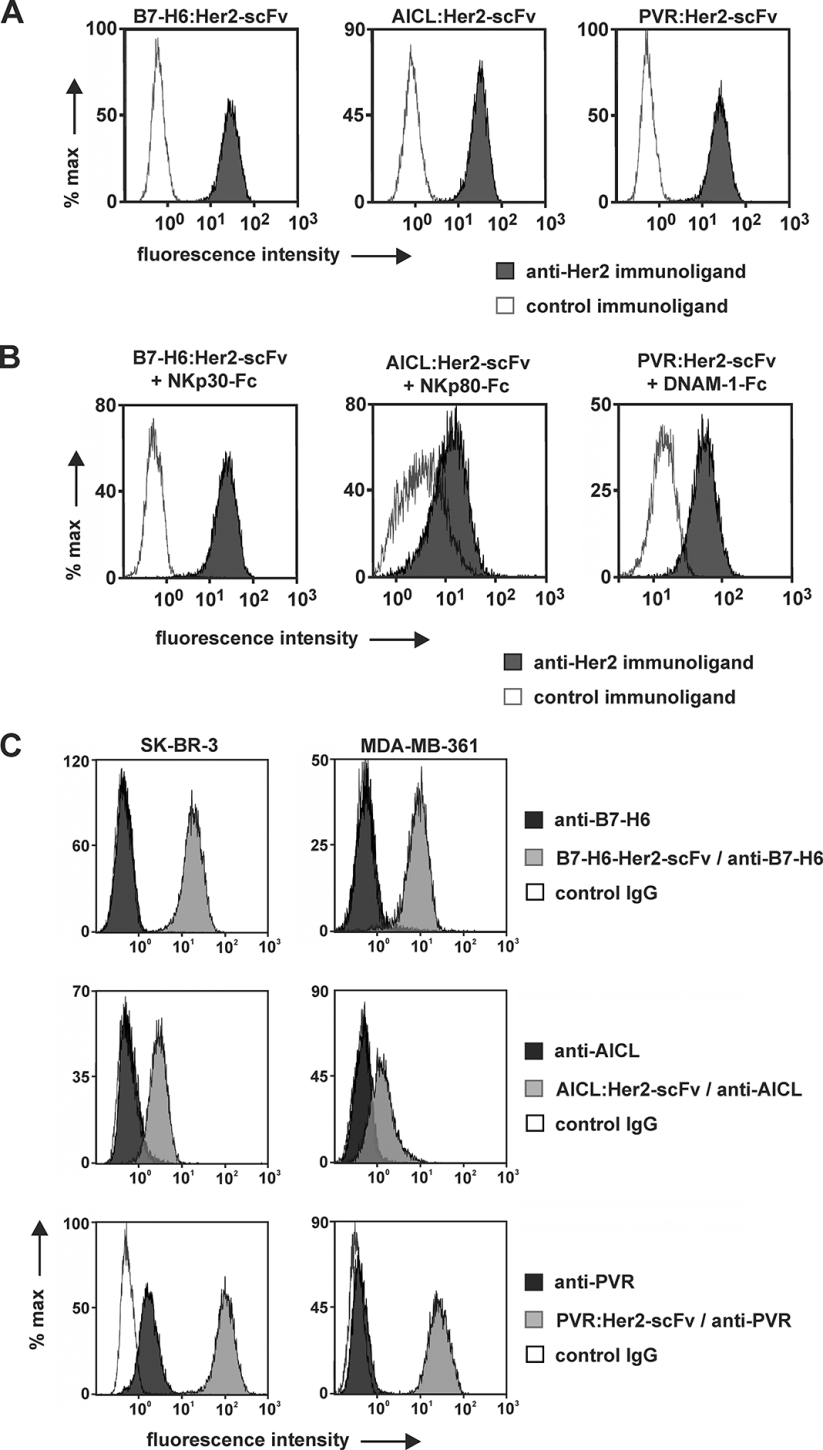
Binding activities of immunoligands **A.** Binding to HER2-positive tumor cells. SK-BR-3 cells were incubated with B7-H6:HER2-scFv, AICL:HER2-scFv, PVR:HER2-scFv or the corresponding control proteins at a concentration of 50 μg/ml. Cell-bound immunoligands were detected with fluorescence labeled anti-penta histidine antibodies and flow cytometry. **B.** Simultaneous antigen binding. SK-BR-3 cells were first incubated with the B7-H6:HER2-scFv or the corresponding control immunoligand and then reacted with a recombinant fusion protein consisting of the ECD of NKp30 and a human IgG Fc domain. Binding was visualized by a fluorescence-coupled antibody against human Fc. Likewise, AICL:HER2-scFv and PVR:HER2-scFv were analyzed for simultaneous antigen binding abilities employing receptor-Fc fusion proteins carrying the ECD of NKp80 and DNAM-1, respectively. **C.** Increased surface levels of alert signals by coating tumor cells with immunoligands. Amounts of ligands endogenously expressed by either SK-BR-3 or MDA-MB-361 cells were compared to surface levels achieved by attaching B7-H6:HER2-scFv, AICL:HER2-scFv and PVR:HER2-scFv. Antibodies specific for B7-H6, AICL and PVR, respectively were employed for detection by flow cytometry. Please note that differences in fluorescence intensities between cells coated with B7-H6:HER2-scFv or AICL:HER2-scFv may be attributed to differences in isotypes of anti-B7-H6 and anti-AICL antibodies and in the detection reagents employed (please refer to materials and methods for further details).

The binding activities of the immunoligands were analyzed with HER2-positive human SK-BR-3 cells and flow cytometry (Fig. 1). B7-H6:HER2-scFv, AICL:HER2-scFv and PVR:HER2-scFv specifically bound to SK-BR-3 cells, whereas no binding was observed with similarly designed control constructs carrying the same ligands but a scFv recognizing the CD37 antigen being not expressed by SK-BR-3 cells (Fig. 1A). As expected, none of the HER2 specific immunoligands reacted with Ramos lymphoma cells not expressing HER2, illustrating specific binding of the immunoconstructs to this antigen (unpublished data). Because binding of the immunoligands to NK cells was hardly detectable by flow cytometry (unpublished data) reactivity with NK cell receptors had to be proven indirectly. To this end, SK-BR-3 cells were first coated with each immunoligand and then incubated with a fusion protein consisting of the extracellular domain of the corresponding NK cell receptors and the human IgG1 Fc domain (i.e. NKp30-Fc, AICL-Fc, PVR-Fc, respectively), which then was detected with a specific, fluorescence-coupled antibody. As a result, NKp30-Fc reacted specifically with SK-BR-3 cells previously coated with B7-H6:HER2-scFv. Likewise, a specific interaction of NKp80-Fc and DNAM-1-Fc with cells coated with either AICL:HER2-scFv or PVR:HER2-scFv, respectively, was demonstrated (Fig. 1B). Therefore, the immunoligands bound both HER2 and the trigger molecule and reacted simultaneously with the target antigen and the soluble form of the NK cell receptor. Thus, B7-H6:HER2-scFv, AICL:HER2-scFv and PVR:HER2-scFv exhibited the proposed bispecific binding abilities. Furthermore, the amounts of endogenously expressed and cell surface displayed B7-H6, AICL and PVR were compared to the surface levels which were achieved by attaching B7-H6:HER2-scFv, AICL:HER2-scFv and PVR:HER2-scFv to the target antigen (Fig. 1C). Only PVR was expressed in significant amounts by SK-BR-3 cells, whereas expression levels of B7-H6 and AICL were below detection limits. However, after incubation with the immunoligands significant amounts of NK cell alert molecules were detectable at the cell surface of these HER2-positive tumor cells.

### Cytotoxic properties of the immunoligands

**Figure 2:**
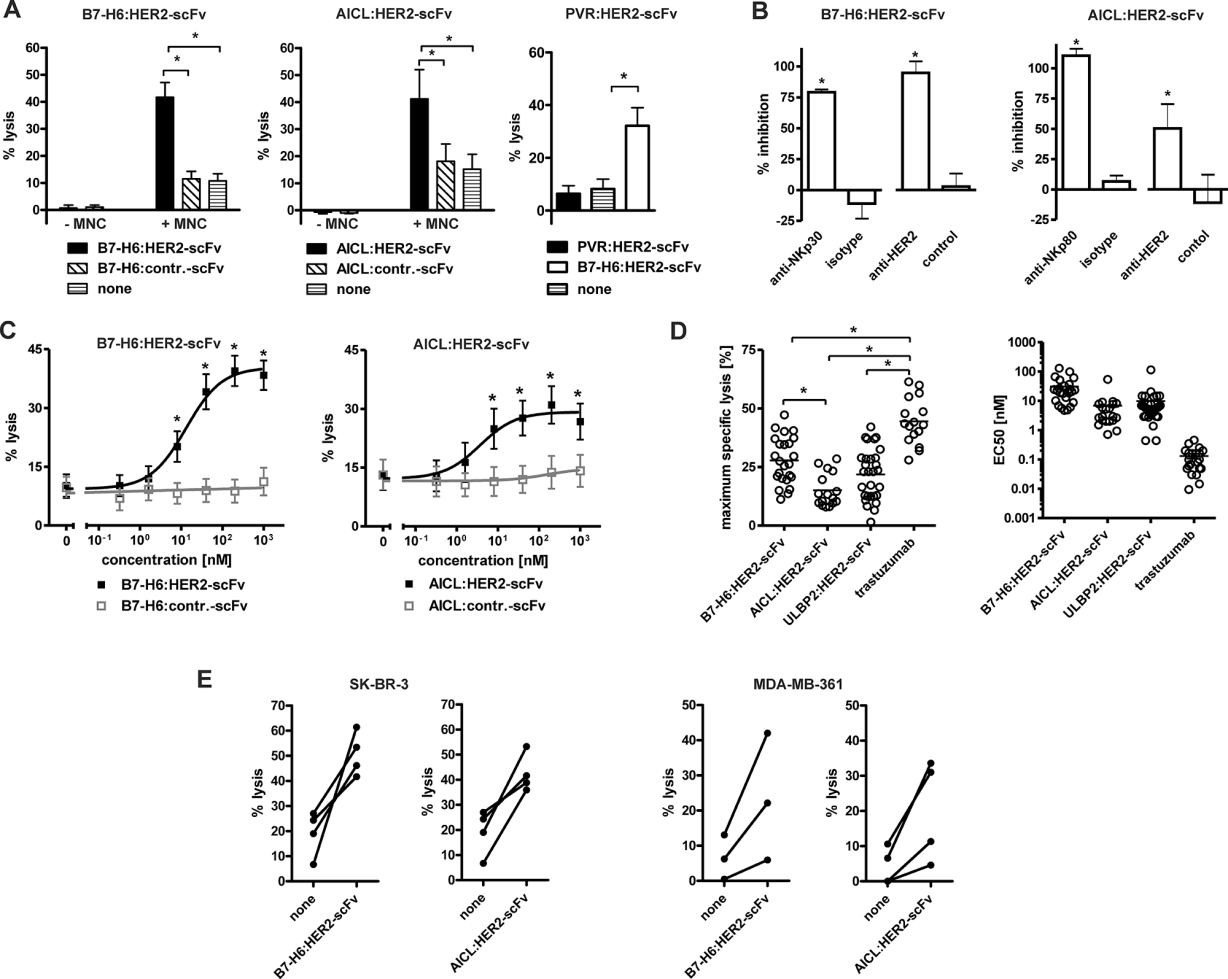
Cytotoxic abilities of the immunoligands **A.** Killing of SK-BR-3 cells by the immunoligands and human MNC effector cells. B7-H6:HER2-scFv and AICL:HER2-scFv at 10 μg/ml mediated lysis of SK-BR-3 cells with human MNCs at an effector-to-target cell (E:T) ratio of 80:1 whereas PVR:HER2-scFv had no lytic effects. No killing was observed when the immunoligands were applied in the absence of MNC or when similar constructed control constructs were applied. Data represent mean values from at least four experiments. Statistically significant differences between groups are indicated (*P < 0.05). **B.** Antigen specific induction of cytotoxicity. Cytotoxicity induced by B7-H6:HER2-scFv and AICL:HER2-scFv was abrogated by addition of murine antibodies against NKp30 and NKp80, respectively, or an antibody-derivative in the tribody format carrying two HER2-specific scFv fragments. The addition of corresponding isotype antibodies or a control tribody had no significant inhibitory effects. MNC were used as effector cells at an E:T ratio of 80:1. Mean values of at least 3 experiments are depicted (*statistically significant differences in comparison to the corresponding control groups, *P* < 0.05). **C.** Effectiveness of B7-H6:HER2-scFv (*n* = 18) and AICL:HER2-scFv (*n* = 12) to induce effector cell-based cytotoxicity was analyzed at varying concentrations employing SK-BR-3 cells and MNC (E:T: 80:1; *P < 0.05). **D.** Efficacy (left panel) and potency (right panel) of B7-H6:HER2-scFv (*n* = 24) and AICL:HER2-scFv (*n* = 16) in comparison to the NKG2D-directed immunoligand ULBP2:HER2-scFv (*n* = 29) and the therapeutic antibody trastuzumab (*n* = 16). Maximum lysis and EC50 were derived from dose response curve using different MNC donors. Mean values are indicated (*P < 0.05). **E.** Cytotoxicity of B7-H6:HER2-scFv and AICL:HER2-scFv with purified NK cells. Purified NK cells were analyzed as effector cells for the immunoligands using SK-BR-3 and MDA-MB-361 cells as targets (E:T ratio: 10:1). Data points represent mean values of triplicate determinations obtained in individual experiments.

The cytotoxic activities of B7-H6:HER2-scFv, AICL:HER2-scFv and PVR:HER2-scFv were analyzed in chromium release assays (Fig. 2A). Human mononuclear cells (MNC) were employed as a source of NK cells and the HER2-positive cell line SK-BR-3 served as a target. B7-H6:HER2-scFv as well as AICL:HER2-scFv triggered lysis of target cells in the presence of effector cells. Importantly, the control molecules B7-H6:contr.-scFv and AICL:contr.-scFv had no cytotoxic effects, indicating that both B7-H6 and AICL required the interaction with a surface antigen expressed by the target cells to trigger the effector cells. In the absence of MNC, however, target cell lysis was neither induced by B7-H6:HER2-scFv nor AICL:HER2-scFv, indicating that effector cell-mediated cytotoxicity was the underlying killing mechanism. In contrast, no cytotoxic effects were observed in reactions containing PVR:HER2-scFv, neither in the absence nor in the presence of MNC (Fig. 2A). Due to the lack of activity of PVR:HER2-scFv, only B7-H6:HER2-scFv and AICL:HER2-scFv were analyzed in more detail in further experiments.

To assess the proposed specific mode of action of the immunoligands blocking experiments were performed, in which either the effector molecule or the HER2 target antigen were masked by antibodies or antibody-derivatives (Fig. 2B). Thus, lysis mediated by B7-H6:HER2-scFv was almost completely blocked by addition of either a murine NKp30-specific antibody or an anti-HER2 tribody, an antibody-based fusion protein carrying two HER2 combining sites. Likewise, cytotoxicity by AICL:HER2-scFv was impaired significantly in the presence of an NKp80-specific antibody or the anti-HER2 tribody. Because isotype control antibodies and a control tribody had no effects, both B7-H6:HER2-scFv and AICL:HER2-scFv required interaction with the target antigen HER2 and engagement of the corresponding trigger molecule (i.e. NKp30 and NKp80, respectively) to induce NK cell-mediated tumor cell lysis.

The killing efficacy of the immunoligands was assayed at varying concentrations using MNC from several different donors. Both B7-H6:HER2-scFv and AICL:HER2-scFv triggered lysis of SK-BR-3 (Fig. 2C) and MDA-MB-361 (Suppl. Fig. 2A) breast cancer cells in a dose-dependent manner and at nanomolar concentrations. SK-BR-3 cells, which express higher levels of HER2 (data not shown), were more sensitive to cytotoxicity induced by the immunoligands, but also were in general more susceptible to NK cell-mediated lysis, also in the absence of any sensitizing antibody construct. Each immunoligand was active at nanomolar concentrations, although both maximum specific lysis achieved at saturating concentrations and half-maximum effective concentrations varied significantly between experiments with effector cells from different donors (Fig. 2D). Overall, B7-H6:HER2-scFv and AICL:HER2-scFv exerted comparable efficacies, with B7-H6:HER2-scFv being slightly more potent in terms of maximum killing but exerting higher EC50 values. The cytotoxic activities were comparable to those observed for another immunoligand, ULBP2:HER2-scFv, engaging the NKG2D receptor. However, none of the immunoligands reached the efficacy of the humanized HER2-specific IgG1 antibody trastuzumab (Fig. 2D).

To demonstrate that the immunoligands triggered NK cells, NK cells from different donors were purified by negative selection by MACS technology and analyzed instead of MNC as effector cells for B7-H6:HER2-scFv and AICL:HER2-scFv in chromium release experiments (Fig. 2E; Suppl. Fig. 2B). As expected, each immunoligand triggered NK cell-mediated lysis of both SK-BR-3 and MDA-MB-361 cells in the presence of purified NK cells, suggesting that NK cells indeed were a relevant effector cell population for both immunoligands.

Both B7-H6:HER2-scFv and AICL:HER2-scFv are glycosylated proteins. To analyse the impact of glycosylation on cytotoxic effects mediated by the immunoligands, deglycosylated variants of both immunoligands were generated by enzymatic digestion under native conditions (Suppl. Fig. 3A). Efficient deglycosylation under these conditions was verified by Western blot analysis. No differences in binding to HER2 between deglycosylated and untreated variants of B7-H6:HER2-scFv and AICL:HER2-scFv were observed (Suppl. Figure 3B). Interestingly, whereas deglycosylation of B7-H6:HER2-scFv had no impact on the molecule's efficacy in mediating tumor cell lysis, the deglycosylated variant of AICL:HER2-scFv was even more effective and was active at lower concentrations (Suppl. Fig. 3C).

**Figure 3:**
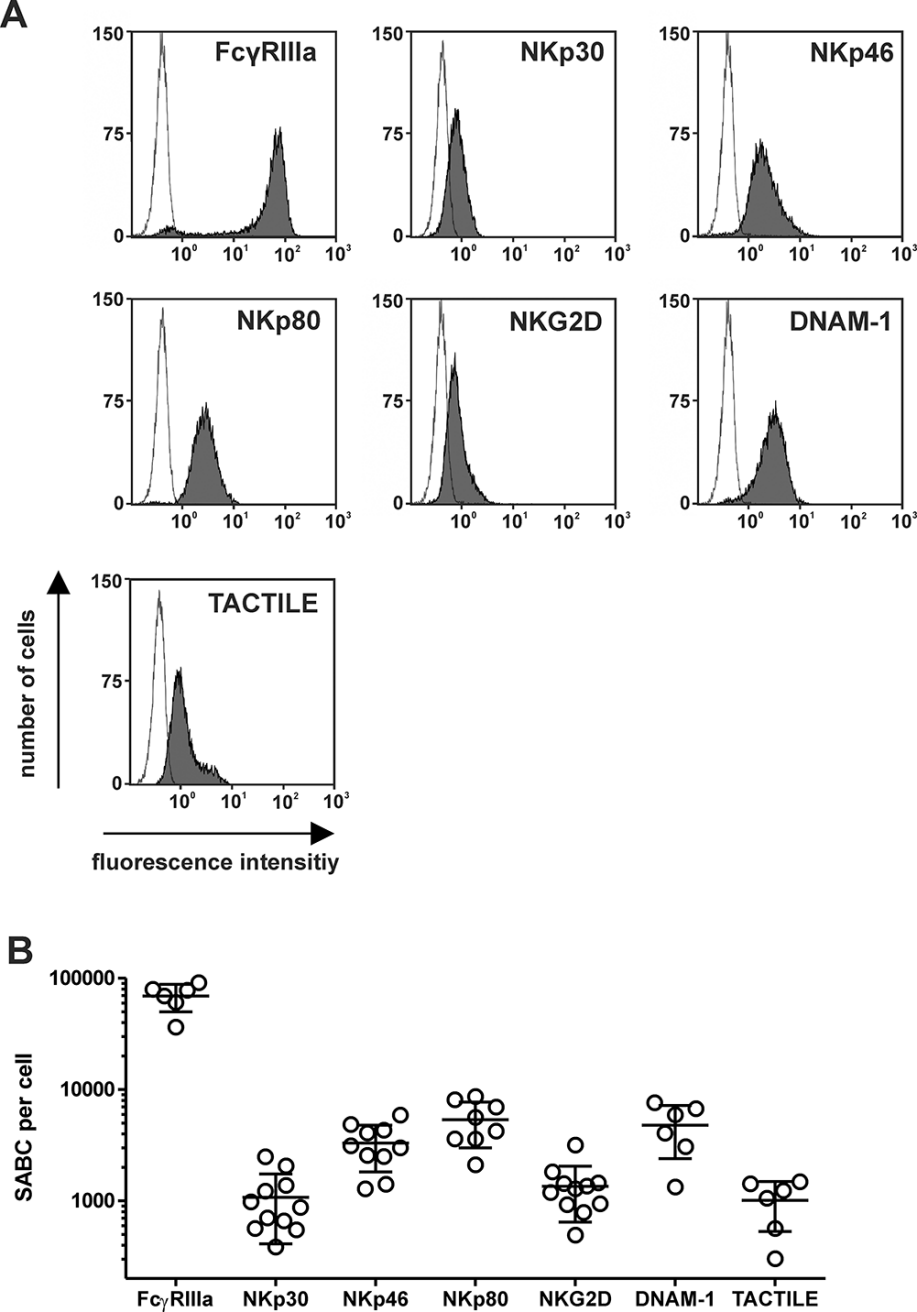
Expression levels of NK cell receptors **A.** Surface expression of activating surface receptors by NK cells was analyzed with specific murine antibodies of the indicated specificities (grey) at saturating concentrations or an isotype control antibody (black line) and flow cytometry. Representative experiments are shown. **B.** Specific antibody binding capacity (SABC) per cell was determined by calibrated flow cytometry using NK cells from different healthy donors. Mean values ± SEM are indicated.

Because the expression levels of NK cell receptors may influence the cytotoxic activity of the immunoligands, the expression of different NK cell receptors was analyzed by calibrated flow cytometry, and SABC per cell were determined as a measure of the cell surface numbers of the receptors (Fig. 3A, 3B). Hence, with exclusion of the minority of the FcγRIIIa-negative CD56^high^ NK cell subpopulation, FcγRIIIa was found abundantly expressed with 70,000 molecules per cell, whereas NKp30, NKp80, DNAM-1 and TACTILE were expressed at significantly lower levels of between 1,000 (NKp30) and 5,000 (NKp80) molecules per cell. Interestingly, despite 5-fold lower expression levels of NKp30, B7-H6:HER2-scFv was more efficacious than AICL:HER2-scFv, suggesting that additional factors are important.

### Synergistic effects by combinations of different immunoligands and enhancement of ADCC

**Figure 4:**
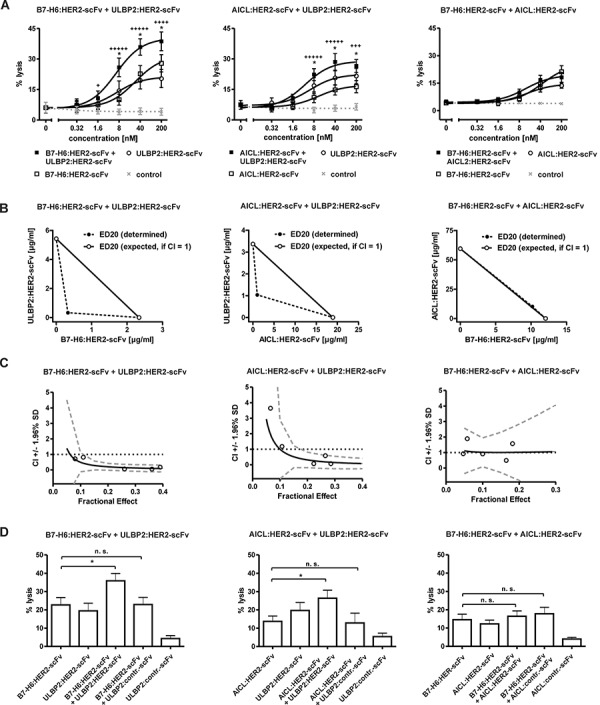
Cytotoxic effects induced by combinations of immunoligands **A.** Dose effect curves for combinations between B7-H6:HER2-scFv and ULBP2:HER2-scFv (left panel), AICL:HER2-scFv and ULBP2:HER2-scFv (middle panel) as well as B7-H6: HER2-scFv and AICL:HER2-scFv (right panel). The effects obtained in combination were compared to effects achieved by the single molecules at varying concentrations. SK-BR-3 cells were used as target cells and MNC served as a source of effector cells. Data points represent mean values ± SEM from at least four independent experiments. Synergistic effects are indicated according to their strength (+++++, CI < 0.1, ++++, CI < 0.3; +++, CI < 0.7). Statistically significant differences between effects by combinations or by the immunoligands as single agents are indicated (*P < 0.05). **B.** Analysis of synergy by isobolograms. The experimentally determined doses resulting in 20% (ED20) target cell lysis were compared with the calculated doses which were expected if additive effects were assumed (CI = 1). **C.** CI values at varying fractional effects. Actual combination data points (open circles) represent mean values of at least four independent experiments [black line, computer simulated CI values; dashed grey line, SD; dotted black line, line of additivity (CI = 1)]. **D.** Enhanced tumor cell lysis was not achieved when HER2-specific immunoligands were combined with corresponding control proteins. Data points represent mean values ± SEM of at least four independent experiments. (*P < 0.05; n.s., not significant).

NK cells are able to integrate activating signals transmitted via different receptors [6]. Thus, to investigate whether NK cell cytotoxicity might be improved by combining immunoligands that engage different activating NK cell receptors, the effects mediated by pairwise combinations of B7-H6:HER2-scFv, AICL:HER2-scFv and ULBP2:HER2-scFv were analyzed employing MNC effector and SK-BR-3 target cells (Fig. 4). As a result, tumor cell lysis was significantly enhanced when B7-H6:HER2-scFv was combined with ULBP2:HER2-scFv relative to lysis obtained with the single agents (Fig. 4A). Calculation of combination index (CI) values and isobologram analysis as a test for synergy, additivity or antagonism indicated that the two immunoligands synergized (Fig. 4B, 4C). At distinct effect levels, determined CI values were even less than 0.1 revealing strong synergistic effects. Also AICL:HER2-scFv and ULBP2:HER2-scFv acted synergistically, although the effects were less pronounced. However, no further enhancement of tumor cell lysis was achieved when B7-H6:HER2-scFv was combined with AICL:HER2-scFv, although at low concentrations additive effects (CI = 1) were observed. Thus, synergistically enhanced tumor cell killing was obtained by concomitant engagement of either NKp30 and NKG2D or NKp80 and NKG2D, whereas co-ligation of NKp30 and NKp80 resulted in additive effects. For comparison, both B7-H6:HER2-scFv and AICL:HER2-scFv were also combined with the corresponding control immunoligands (Fig. 4D). However, in this setting the extent of killing was not further increased, indicating that antigen-specificity was maintained when the different immunoligands were used in combination.

**Figure 5:**
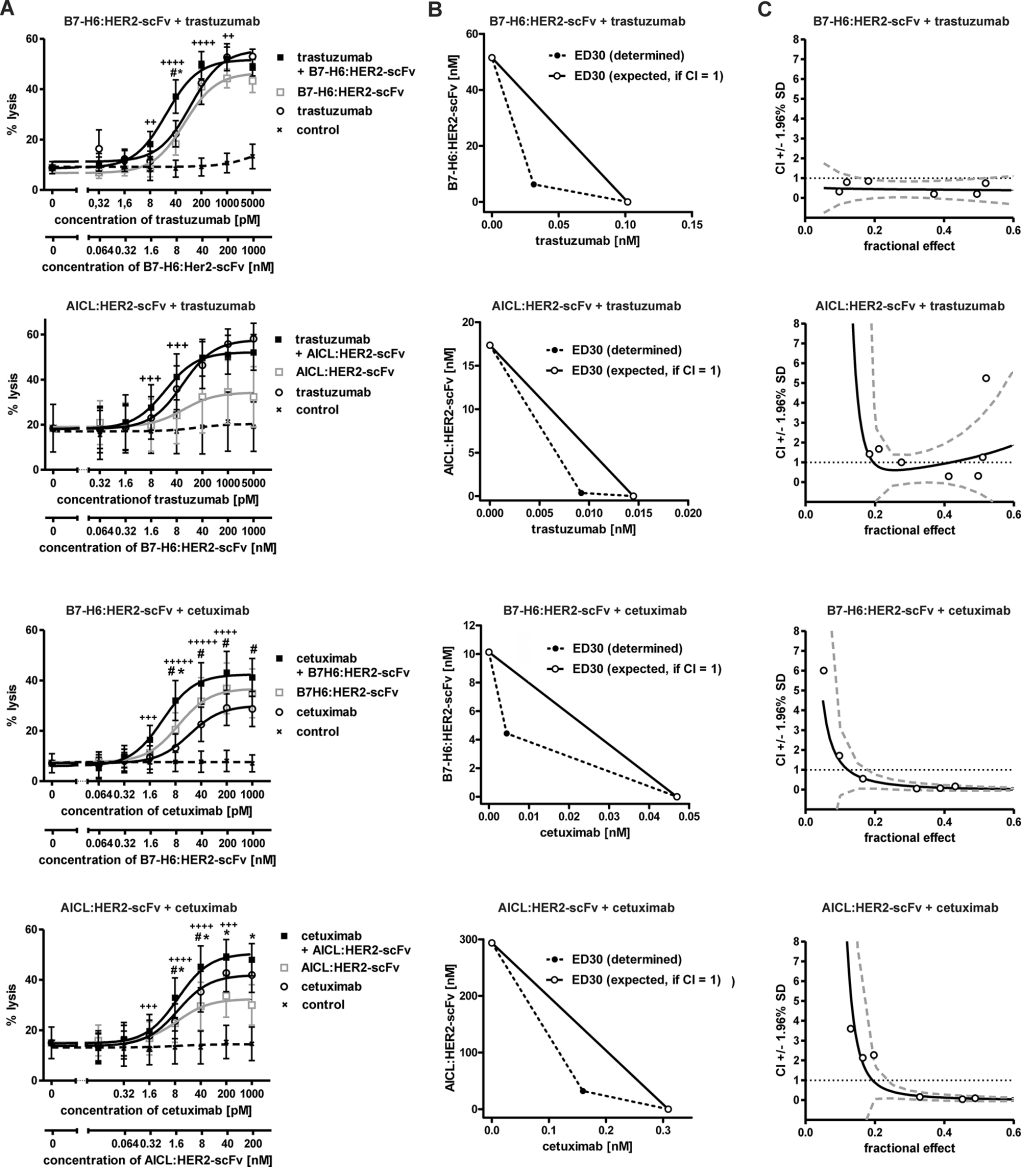
Cytotoxic effects induced by combinations of immunoligands and therapeutic antibodies **A.** Dose effect curves for pairwise combinations between the immunoligands and the therapeutic antibodies trastuzumab and cetuximab. The effects obtained in combination were compared to the effects achieved by the single molecules at varying concentrations. SK-BR-3 cells were employed as target cells and MNC served as an effector cell source. Rituximab was employed as an IgG1 control antibody. Data points represent mean values ± SEM from at least four independent experiments. Synergistic effects are indicated according to their strength (+++++, CI < 0.1, ++++, CI < 0.3; +++, CI < 0.7; ++, CI < 0.85). Statistically significant differences between effects induced by combinations or the immunoligands (#*P* < 0.05) or by the antibodies as single agents (*P < 0.05) are indicated. **B.** Analysis of synergy by isobolograms. The experimentally determined doses resulting in 30% (ED30) target cell lysis were compared with the calculated doses which were expected, if additive effects were assumed (CI = 1). **C.** CI values at varying fractional effects. Actual combination data points (open circles) represent mean values of at least four independent experiments [black line, computer simulated CI values; dashed grey line, SD; dotted black line, line of additivity (CI = 1)].

ADCC represents an important effector function for many therapeutic antibodies [9, 32]. Therefore we were interested whether B7-H6:HER2-scFv and AICL:HER2-scFv were able to boost ADCC, as it has been suggested previously for other immunoligands and individual antibodies [29, 30]. To this end, B7-H6:HER2-scFv and AICL:HER2-scFv were analyzed in combination with the HER2-specific antibody trastuzumab (Fig. 5A). Whereas B7-H6:HER2-scFv enhanced ADCC by trastuzumab significantly in a synergistic manner (Fig. 5B, 5C), no relevant increases in ADCC were obtained in the presence of AICL:HER2-scFv (although mathematical testing revealed synergy also for this combination). Moreover, to analyze an antibody as a combination partner that recognized a target antigen different from that bound by the immunoligands, B7-H6:HER2-scFv and AICL:HER2-scFv were combined with cetuximab targeting the epidermal growth factor receptor (EGFR, Fig. 5A). EGFR is co-expressed with HER2 by SK-BR-3 and MDA-MB-361 cells, although at significantly lower levels (Suppl. Figure 4A, Suppl. Figure 5A). In agreement with results obtained with trastuzumab, B7-H6:HER2-scFv boosted cetuximab-mediated ADCC against SK-BR-3 (Fig. 5A) and MDA-MB-361 (Suppl. Fig. 5B) cells synergistically. Furthermore, significant increases in target cell lysis were also achieved by combining the antibody with AICL:HER2-scFv (Fig. 5A, Suppl. Fig. 5B). However, the efficacy of the combination of B7-H6:HER2-scFv and cetuximab was not reached. Of note, antigen-dependence was maintained when the antibody and the immunoligands were applied in combination, because cytotoxicity was not observed when an isotype control antibody was combined with a control immunoligand (Suppl. Fig. 4B). Moreover, ADCC induced by cetuximab was not enhanced when the corresponding control immunoligands were added (Suppl. Fig. 4B). Thus, NK cell cytotoxicity was triggered in a synergistic manner by combining therapeutic antibodies with B7-H6:HER2-scFv, and, to a lesser extent, with AICL:HER2-scFv, suggesting a cooperation of NKp30, NKp80 and FcγRIIIa in NK cell activation and cytotoxicity.

## DISCUSSION

With the aim to increase the visibility of tumor cells to NK cells, three bispecific immunoligands carrying a HER2-specific scFv and the ECD of B7-H6, AICL or PVR were generated to trigger NK cells via the activating receptors NKp30, NKp80 or DNAM-1, respectively. Whereas PVR:HER2-scFv did not mediate cytotoxic effects, both B7-H6:HER2-scFv and AICL:HER2-scFv promoted NK cell cytotoxicity and induced killing of HER2-positive tumor cells. B7-H6:HER2-scFv as well as AICL:HER2-scFv synergized with the immunoligand ULBP2:HER2-scFv engaging NKG2D and each were able to boost ADCC by monoclonal antibodies, suggesting that both NKp30 and NKp80 may represent attractive effector molecules on NK cells for the design of immune-modulatory biological agents.

Raising NK cells against tumors may be a promising treatment option for various types of cancer [3]. Several strategies have been followed with the aim to promote the anti-tumoral effector functions of NK cells. Thus, NK cell cytotoxicity was for example enhanced by small molecules, interleukins as well as antibodies or antibody-derivatives [3]. Recombinant immunoligands such as B7-H6:HER2-scFv and AICL:HER2-scFv are bispecific antibody-based biologics equipped with a targeting device, which may allow tumor cell–specific activation of NK cells [26]. Moreover, such molecules offer the opportunity to trigger NK cell effector functions with antibody-based molecules in an Fc receptor independent manner. By engagement of other surface receptors it may be assumed that the immunoligands may provoke a different shape of immune response as monoclonal IgG antibodies, which needs to be addressed in further studies. Importantly, AICL:HER2-scFv, which to our knowledge represents the first biological agent that was designed to attract NK cells via NKp80, was able to trigger tumor cell lysis. These results provide proof of concept that NKp80 may - in addition to NKG2D and NKp30 - be considered as a valuable trigger molecule on NK cells for recombinant immunoligands or other formats of bispecific antibody-derivatives.

The potency of individual immunoligands may reflect the characteristics of the corresponding NK cell receptor. Of note, the NK cell receptors bound by the immunoligands analyzed in this study differed in their intracellular signaling pathways. Whereas NKp30 similar to FcγRIIIa is coupled to the immunoreceptor tyrosine-based activation motif (ITAM)-bearing CD3ζ or FcεRIγ chains, NKp80, DNAM-1 and NKG2D function ITAM-independent [5, 6]. Thus, NKp80 contains a hemi-ITAM-like domain [33], DNAM-1 associates upon engagement with the integrin lymphocyte function-associated antigen-1 and contains two phosphorylation sites in its intracellular part [34], whereas NKG2D pairs with the adaptor molecule DNAX activating protein of 10 kDa containing a tyrosine-based YxxM motif, different form the ITAM [6, 35]. We surmise that the intracellular signaling pathway may critically influence the efficacy of the immunoligands and determine the receptor's suitability as a trigger molecule for antibody-based approaches. Furthermore, this may explain the observed differences in the efficacy between individual immunoligands or between the immunoligands and IgG1 antibodies. However, additional factors may be critical. For example, the expression levels of the receptors may influence the cytotoxic activity of the immunoligand. Of note, the surface amounts of receptors controlling natural cytotoxicity were significantly lower than surface amounts of FcγRIIIa, which upon engagement by an antibody produced the strongest cytotoxic effects. In general, the cytotoxic properties of the immunoligands may be limited by monovalent tumor cell binding and the low binding affinity to the NK cell receptor. Together with lower expression levels and differences in signaling properties, this may in part explain why the immunoligands did not reach the efficacy of trastuzumab, which carries identical antibody V-regions. This limitation may be overcome by further optimizing the format. For example, increasing the valence by converting the immunoligands into bivalent dimers or trivalent trimers may significantly enhance these immunoligands’ cytotoxic activities, as it has been shown for other immunoligands carrying ULBP2 [36]. Also the generation of bispecific antibodies with NKp30 or NKp80 combining sites may offer the opportunity to recruit NK cells via these receptors with an enhanced efficacy due to an increased binding affinity.

The lack of activity of PVR:HER2-scFv may be attributed to a weak signal transmitted by engagement of the receptor, an unfavorable cell surface distribution of the HER2-bound immunoligand, or the strict requirement for engagement of additional activating or co-activating receptors. Moreover, also the recognition of additional NK cell receptors may play an important role. Whereas B7-H6, AICL and ULBP2 each ligate a single NK cell receptor, PVR is recognized not only by DNAM-1, but also by T cell activation increased late expression (TACTILE, CD96) and T cell Ig and ITIM domain (TIGIT), which both are expressed by NK cells as well. Whereas the adhesion molecule DNAM-1 promotes NK cell cytotoxicity [37], TIGIT provides inhibitory effects [38], and for TACTILE both activating and inhibitory functions have been reported [39, 40]. Thus, the lack of efficacy by PVR:HER2-scFv may in part be due to a competition between three receptors with partially opposed functions [40]. Along this line TACTILE has been shown to bind PVR with greater affinity than DNAM-1 [41]. Thus, on NK cells PVR was shown to predominantly bind to TACTILE, which might impair an efficient activation of NK cells [40].

Importantly, synergistic effects were achieved when distinct immunoligands were combined to engage different NK cell receptors simultaneously. Of note, synergistic effects were achieved despite cross-competitive binding to the same HER2 epitope. However, not all combinations lead to increased tumor cell lysis and no significant improvements were obtained when B7-H6:HER2-scFv was combined with AICL:HER2-scFv. Thus, synergy was observed for the combination between B7-H6:HER2-scFv and ULBP2:HER2-scFv as well as between AICL-HER2-scFv and ULBP2:HER2-scFv. These findings indicate cooperation between NKG2D and NKp30 or NKp80 in triggering NK cell cytotoxicity and are in agreement with our previous results obtained with CD20-directed immunoligands containing either ULBP2 or B7-H6 [30]. The synergistic effects may depend on the induction of different intracellular signaling pathways, stabilization of the immunological synapse, or by the magnitude of the activating signal. Moreover, both B7-H6:HER2-scFv and, although to a lesser extent, AICL:HER2-scFv were able to boost NK cell-mediated ADCC by therapeutic antibodies, which targeted either the same or another target antigen expressed on tumor cells. Enhancing ADCC by NK cells, which is anticipated to be an important mechanism of many therapeutic antibodies, represents an interesting option to improve antibody therapy [4, 7, 8, 42]. In various combination strategies ADCC was enhanced by combining the antibody with varying drugs, antibodies or other biologics. However, previous efforts to enhance ADCC by combining two IgG antibodies targeting tumor cell antigens have failed [29, 43, 44], presumably because the two antibodies mutually cross-blocked Fc receptor binding. Thus, Fc receptor independent antibody-derivatives such as the bispecific immunoligand B7-H6:HER2-scFv may provide an attractive alternative to be employed in antibody combination strategies.

The induction of synergistic effects by engagement of different NK cell receptors along with the possibility to target two different target antigens may even allow preferential killing of tumor cells, given that malignant cells express both target antigens at significant densities while healthy cells do not. In this setting, the selection of appropriate target antigens will be critical to realize this “dual-dual targeting” concept and further studies are warranted to provide proof-of-concept.

In conclusion, B7-H6:HER2-scFv and AICL:HER2-scFv exerted cytotoxic abilities as single agents and in combination with NKG2D-specific immunoligands or monoclonal antibodies. In particular with their abilities to boost ADCC by therapeutic antibodies in a synergistic manner these immunoligands represent interesting novel immunomodulatory biologics designed for cancer immunotherapy.

## MATERIALS AND METHODS

### Cell culture

The human breast adenocarcinoma cell lines SK-BR-3 and MDA-MB-361 (American Type Culture Collection) were cultured in McCoy's 5a medium (Invitrogen) supplemented with 20% heat inactivated fetal calf serum (FCS; Invitrogen), 100 units/ml penicillin (Invitrogen) and 100 μg/ml streptomycin (Invitrogen) and in Dulbecco's Modified Eagle's Medium (DMEM, Invitrogen) containing 20% FCS, 100 units/ml penicillin and 100 μg/ml streptomycin, respectively. Lenti-X 293T cells (Takara Bio Europe / Clontech) were maintained in DMEM supplemented with 10% FCS, 100 units/ml penicillin and 100 μg/ml streptomycin.

### Preparation of MNC and isolation of NK cells

Experiments were approved by the Ethics Committee of the Christian-Albrechts-University (Kiel, Germany), in accordance with the Declaration of Helsinki. Blood was drawn after receiving the donors’ informed consent. MNC were prepared as described previously [45]. NK cells were isolated by MACS technology by negative selection using NK cell isolation kit according to the manufacturer's protocol (Miltenyi). Purified NK cells were cultured over-night at a density of 2 × 10^6^ cells/ml in RPMI 1640 Glutamax-I medium supplemented with 10% FCS, 100 units/ml penicillin and 100 μg/ml streptomycin.

### Generation of immunoligands

The variable regions derived from the humanized antibody humAb4D5-8 were employed for the generation of HER2-specific immunoconstructs [46, 47]. The construction of derivatives of the pSecTag2 HygroC vector (Invitrogen) for expression of B7-H6:HER2-scFv and ULBP2:HER2-scFv has been described previously [30]. The sequences of the extracellular domains of AICL (amino acids 26 – 149) or PVR (amino acids 1 – 339) were synthesized *de novo* according to published sequences (Eurofins MWG GmbH) [19, 48]. The expression vector encoding PVR:HER2-scFv was constructed by exchanging the sequences for ULBP2 against those encoding the ECD of PVR. For construction of AICL:HER2-scFv, the cDNA encoding the extracellular domain of AICL was cloned into a derivative of the Strep Tag II vector (Invitrogen) containing a BM40 signal peptide and 5′-terminal Strep and hexa-histidine tags (unpublished data). Finally, the HER2-specific scFv 4D5-8 was ligated to the 5′ end of AICL. The expression vectors encoding the control immunoligands were generated by replacing the coding sequences for scFv 4D5-8 by those encoding a CD37 scFv, which had been synthesized *de novo* according to published sequences [49]. The immunoligands were expressed transiently in Lenti-X 293T cells by calcium-phosphate transfection (Invitrogen) and purified by affinity chromatography using nickel-nitrilotriacetic acid (Ni-NTA) agarose beads (Qiagen) as described earlier [30]. Concentrations of purified proteins were estimated against a standard curve of BSA or determined by quantitative capillary electrophoresis using Experion™ Pro260 technology (BioRad) in accordance with the manufacturer's protocol.

### Antibodies and antibody-derived fusion proteins

Murine antibodies from hybridomas 4D5 (anti-HER2, ATCC), 225 (anti-EGFR, ATCC) and TH-111 (anti-TACTILE; CD96) [50] were purified from cell culture supernatants by standard procedures using protein A beads (Sigma-Aldrich). The therapeutic antibodies trastuzumab, rituximab (both from Roche Pharma AG) and cetuximab (Merck) were purchased. Antibodies specific for NKG2D (murine IgG1), NKp30 (murine IgG2a), NKp80 (rat IgG2a) and DNAM-1 (murine IgG1) were obtained from R&D Systems. Mouse IgG1 antibodies specific either for FcγRIII or NKp80 were purchased from Santa Cruz and Miltenyi, respectively. B7-H6 was detected using polyclonal anti-B7-H6 IgG and secondary, PE-conjugated polyclonal donkey anti-rabbit IgG F(ab’)_2_ (each from Abcam). PVR was detected with specific PE-coupled antibodies (R&D systems). Cell surface AICL was visualized with goat polyclonal anti-AICL antibodies and secondary FITC-labeled donkey anti-goat IgG (each from Santa Cruz Biotechnology). All antibodies were applied according to the manufacturer's recommendations and appropriate isotype-matched antibodies were used as controls. The fusion protein containing the ECD of NKp30 and the human IgG Fc domain (NKp30-Fc) was expressed and purified as described previously [30]. NKp80-Fc and DNAM-1-Fc were obtained from R&D Systems. The anti-HER2 tribody (fusion protein between two anti-HER2 scFv and a CD89 Fab fragment; unpublished data) and the control tribody (two anti-EGFR scFv fused to a CD89 Fab; unpublished data) were produced as described previously for other tribody molecules.

### Homology modeling

YASARA Structure software (YASARA Biosciences) was employed to calculate the homology models for the HER2-specific scFv derived from antibody humAb4D5-8 and the ligands B7-H6, AICL and PVR individually. Secretion leader sequences and tags were removed. Structures for whole molecules were generated by introducing linker sequences and fusing the best-fitting models obtained for the single subunits. Ribbon drawings were generated using Discovery Studio 2.0 Visualize software (Accelrys Inc.).

### Sodium dodecyl sulphate-polyacrylamide gel electrophoresis (SDS-PAGE), Western blotting and gel filtration chromatography

SDS-PAGE and Western transfer experiments were performed by standard procedures [30]. The immunoligands were detected applying mouse anti-penta-His (Qiagen) and secondary horse radish peroxidase-conjugated goat anti-mouse IgG antibodies (Dianova). Gel filtration chromatography was performed on an ÄKTA purifier (GE Healthcare) employing Superdex 200 10/300 GL column (GE Healthcare). Phosphate buffered saline (PBS) was used as running buffer at a constant flow rate of 0.7 ml/min. 100–500 μg protein were loaded in a volume of 0.5 ml PBS. Ferritin (440 kDa), human IgG1 (150 kDa), Conalbumin (75 kDa) and Ribonuclease A (13.7 kDa) were used for calibration. Data were analyzed with Unicorn 5.1 software (GE Healthcare).

### Deglycosylation of the immunoligands

Immunoligands were deglycosylated under denaturing reaction conditions using Protein Deglycosylation Mix (New England Bio Labs) containing the enzymes O-glycosidase, PNGase F, neuraminidase, β1–4 galactosidase and β-N-acetylglucosaminidase according to the manufacturer's instructions. For western blotting, 1.5 μg of proteins were loaded onto a gel.

### Flow cytometry

Flow cytometry was performed on a Navios flow cytometer (Beckman Coulter) as described earlier [30]. For binding studies, the immunoligands were applied at a concentration of 50 μg/ml. An Alexa Fluor 488-coupled anti-penta His secondary antibody (Qiagen) was employed for detection. To demonstrate simultaneous binding, SK-BR-3 cells were reacted with the immunoligands at 50 μg/ml and then incubated with an Fc-fusion protein containing the corresponding NK cell receptor (i.e. NKp30-Fc, NKp80-Fc, or DNAM-1-Fc) at 100 μ g/ml and subsequently with polyclonal FITC-coupled anti-human IgG F(ab’)_2_ fragments (Beckman Coulter). Receptor expression levels were quantitated by determination of specific antigen binding capacities (SABC) employing Qifikit (DAKO) according to manufacturer's protocols.

### Analysis of cell-mediated cytotoxicity

Cytotoxicity was analyzed in standard 3 h ^51^Cr release assays performed in 96-well microtiter plates in a total volume of 200 μl as described previously [45]. Human MNC or purified NK cells were used as effector cells at effector-to-target cell (E:T) ratios of 80:1 and 10:1, respectively. The immunoligands and antibodies were applied at the indicated concentrations. For blocking experiments, cells were pre-incubated with masking antibodies or tribodies (each at 50 μg/ml) for 30 min before the immunoligands were added. Values for maximum specific lysis and EC50 of trastuzumab were derived from dose response curves employing varying antibody concentrations up to 750 ng/ml.

### Data processing and statistical analyses

Graphical and statistical analyses were performed with GraphPad Prism 5.0 software. *P*-values were calculated employing repeated measures ANOVA and the Bonferroni post-test, or the student's *t*-test when appropriate. *P* ≤ 0.05 were regarded as statistically significant. CI values were calculated with CalcuSyn software (Biosoft) according to the method of Chou and Talalay using the formula CI_*x*_ = D_A_/D_*x*A_ + D_B_/D_*x*B_ [51], where D_*x*A_ and D_*x*B_ indicate doses of drug A and drug B alone producing *x*% effect, and D_A_ and D_B_ indicate doses of drugs A and B in combination producing the same effect.

## SUPPLEMENTARY FIGURES LEGENDS


