# Supplementary material for: HER2-specific immunoligands engaging NKp30 or NKp80 trigger NK-cell-mediated lysis of tumor cells and enhance antibody-dependent cell-mediated cytotoxicity
Source: Oncotarget. 2015 Sep 15;6(31):32075–88. doi: 10.18632/oncotarget.5135 (PMC4741660; doi:10.18632/oncotarget.5135)
Supplement: Supplementary file 1 [file oncotarget-06-32075-s001.pdf]

SUPPLEMENTAL FIGURES LEGENDS

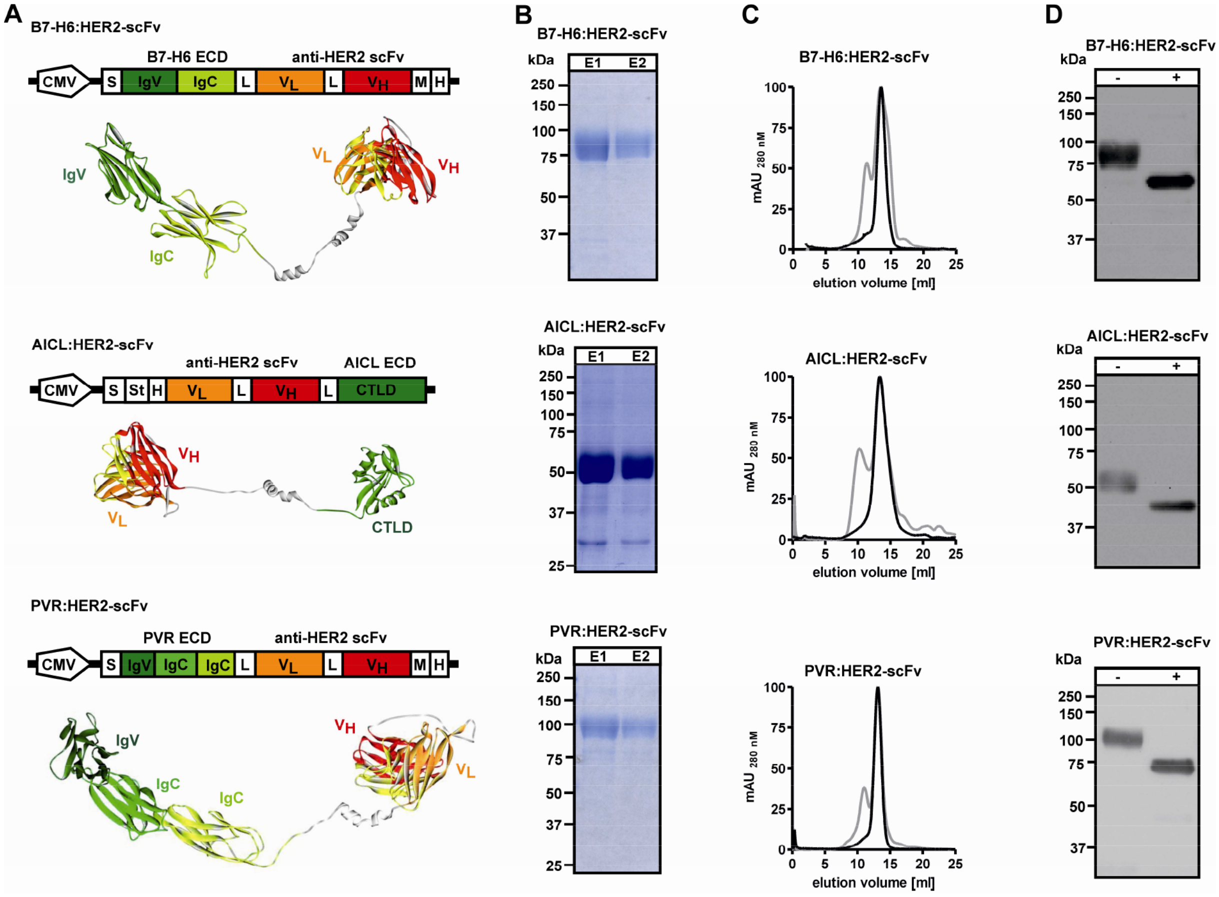

Supplementary Figure S1: Design, expression and binding of recombinant immunoligands. A. Construction and homology models of B7-H6:HER2-scFv (top panel), AICL:HER2-scFv (middle) and PVR:HER2-scFv (bottom). The extracellular domains (ECD) of B7-H6, AICL and PVR (green) were genetically fused to a HER2-specific scFv containing the variable domains of light (V_L, orange) and heavy chains (V_H, red) from the humanized antibody humAb4D5–8. The complementarity determining regions are colored in yellow and linker sequences in grey. Individual V-type Ig-like (IgV) or C-type Ig-like domains (IgC) included in both B7-H6 and PVR as well as the C-type lectin-like domain (CTLD) of AICL are indicated. To maintain the natural orientation of the ligands, the fragment AICL, a type II transmembrane protein, was fused to the C-terminus of the scFv, whereas the two other ligands, which both are type I transmembrane proteins, were ligated to its N-terminus (CMV, cytomegalovirus promotor; S, signal peptide; St, Strep tag II, H, hexa histidine tag; M, c-myc epitope). **B.** After purification by affinity chromatography, the immunoligands were analyzed by SDS gel electrophoresis under reducing conditions and subsequent staining with Coomassie brilliant blue. E1, E2, consecutive elution fractions. **C.** Gel filtration. To remove minor contaminants or potential multimers, Ni-NTA-purified immunoligands (grey) were subjected to gel filtration. Appropriate fractions were collected and re-analyzed (black). **D.** Deglycosylation of immunoligands and analysis by western blotting. B7-H6:HER2-scFv, AICL:HER2-scFv and PVR:HER2-scFv were subjected to enzymatic deglycosylation under denaturing conditions and analyzed by western blotting using mouse anti-penta His antibody for detection (lane 1: denatured immunoligand; lane 2: denatured immunoligand treated with deglycosylation mix). Representative results are shown.

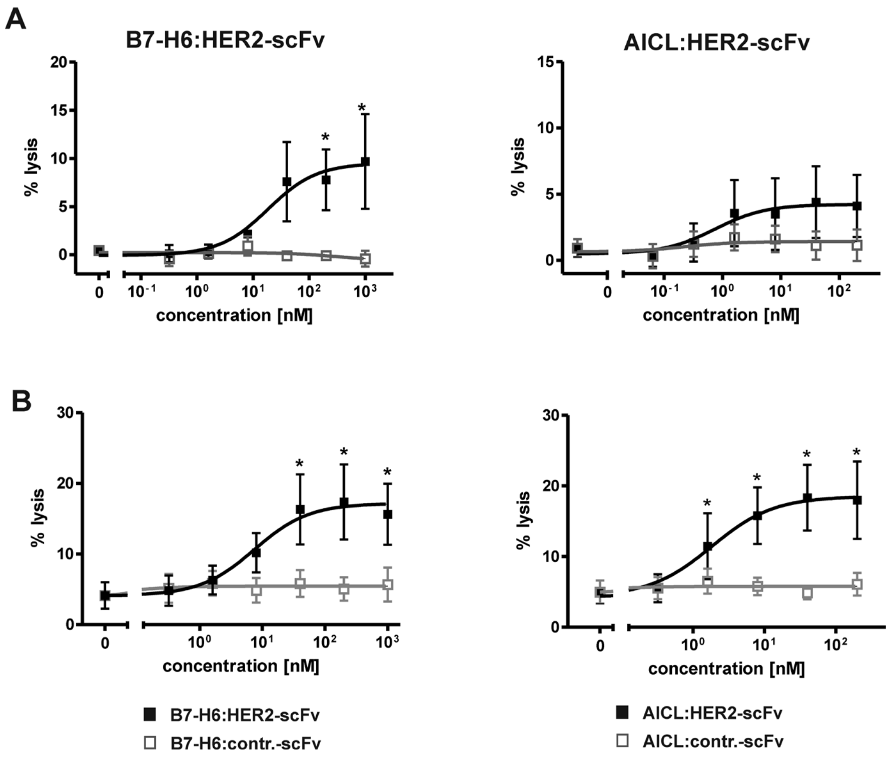

Supplementary Figure S2: Killing of MDA-MB-361cells by the immunoligands. Cytotoxic activities of B7-H6:HER2-scFv and AICL:HER2-scFv were analyzed at varying concentrations using **A.** human MNC or **B.** NK cells as effector cells. Effector-to-target cell (E:T) ratios were of 80:1 and 10:1 for MNC and NK cells, respectively. Data represent mean values from at least four experiments employing effector cell preparations from different healthy individuals. Statistically significant differences between groups are indicated (*$P < 0.05$).

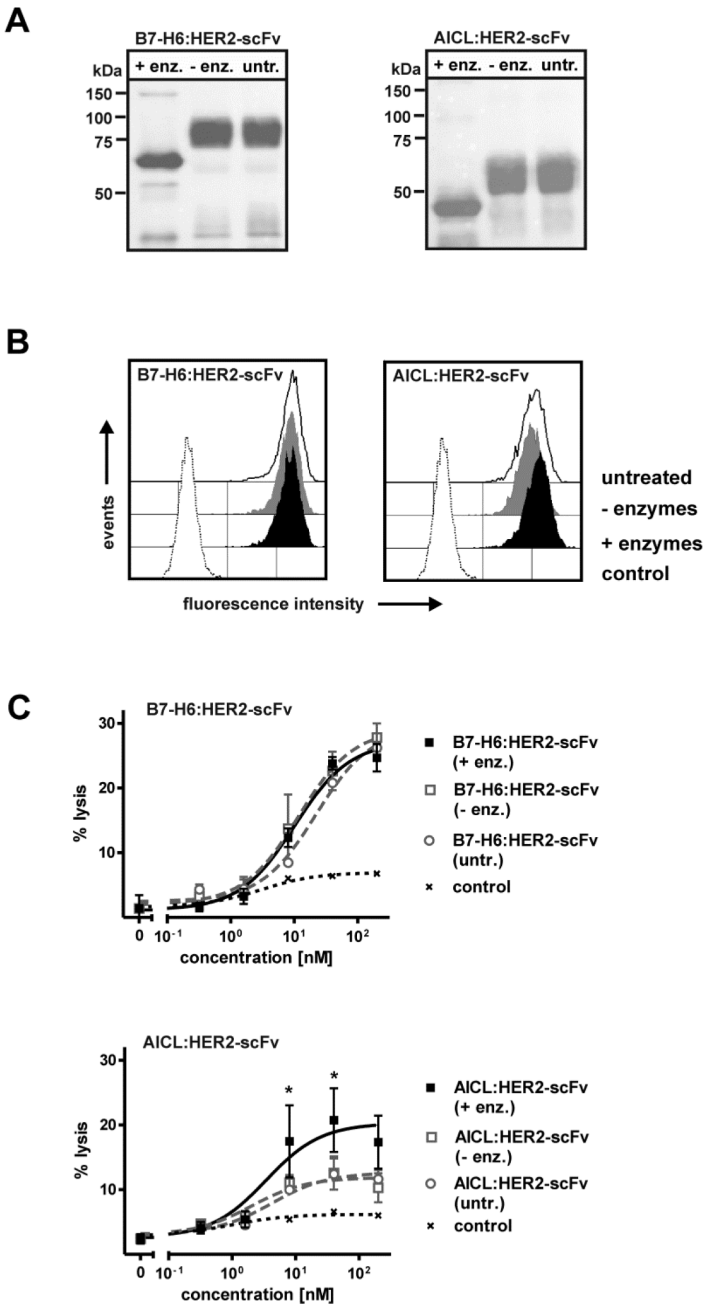

Supplementary Figure S3: Effect of deglycosylation on functions of B7-H6:HER2-scFv and AICL:HER2-scFv. A. Twenty micrograms of the immunoligands were subjected to enzymatic deglycosylation under native conditions. Four micrograms of each immunoligand were analyzed by western blotting using mouse anti-penta His antibody for detection (lane 1: immunoligand treated with deglycosylation mix; lane 2: immunoligand treated with buffer alone; lane 3: untreated immunoligand). One representative experiment is shown ($n = 3$). **B.** The differentially treated immunoligands were analyzed for binding to HER2-positive SK-BR-3 cells by flow cytometry (concentrations were 300 µg/ml; $n = 3$). **C.** In ^{51}Cr release experiments the deglycosylated variants of B7-H6:HER2-scFv and AICL:HER2-scFv (+ enz.) were compared either with the corresponding immunoligands treated with buffer alone (-enz.), with untreated molecules (untr.), or buffer (control). SK-BR-3 cells were employed as target cells and MNC were used as effector cells at an E:T cell ratio of 80:1. Data points represent mean values ± SEM of 3 independent experiments. Statistically significant differences between deglycosylated immunoligands and buffer or untreated proteins are indicated ($*P \leq 0.05$).

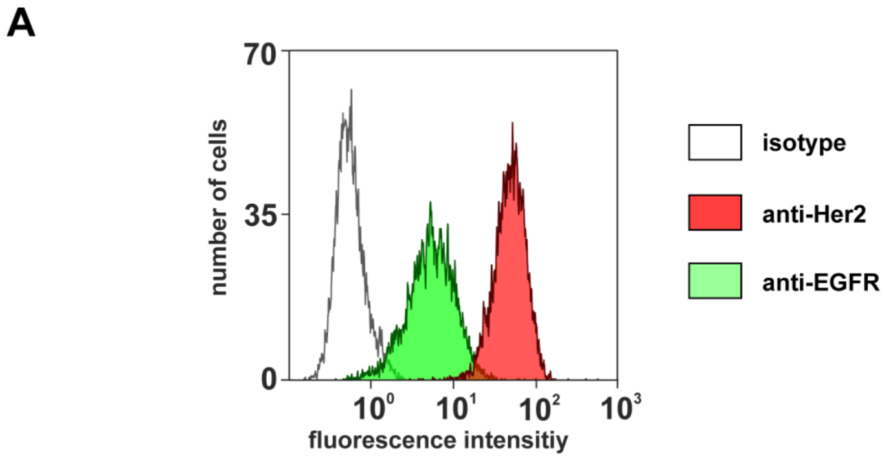

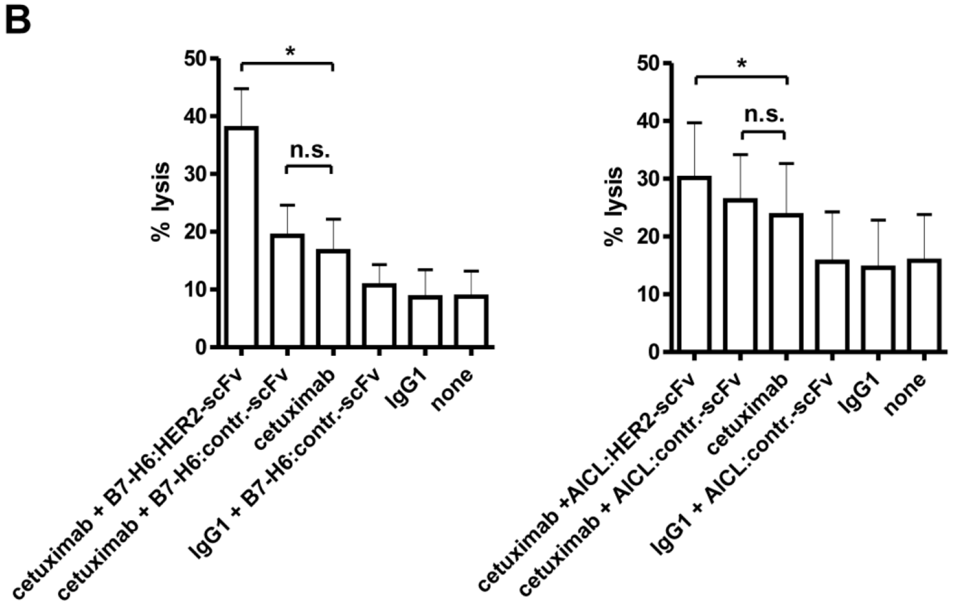

Supplementary Figure S4: Enhancement of ADCC by immunoligands is antigen-specific. A. SK-BR-3 cells co-display HER2 and EGFR. Expression was analyzed using specific antibodies and flow cytometry. **B.** Specificity of the immunoligands in the presence of the EGFR-specific antibody cetuximab. ADCC is enhanced by B7-H6:HER2-scFv (left graph) or AICL:HER2-scFv (right graph), whereas no improvements were obtained when the antibody was combined with the corresponding control immunoligands. Combinations between a control IgG1 antibody (rituximab) and control immunoligands did not trigger tumor cell lysis. B7-H6:HER2-scFv and AICL:HER2-scFv were used at 8 nM and 1.6 nM, respectively. Antibodies were applied at 8 pM. MNC served as effector cells at an E:T cell ratio of 80:1. Data points represent mean values ± SEM of four independent experiments. Statistically significant differences are indicated (**P* < 0.05; n.s., not significant).

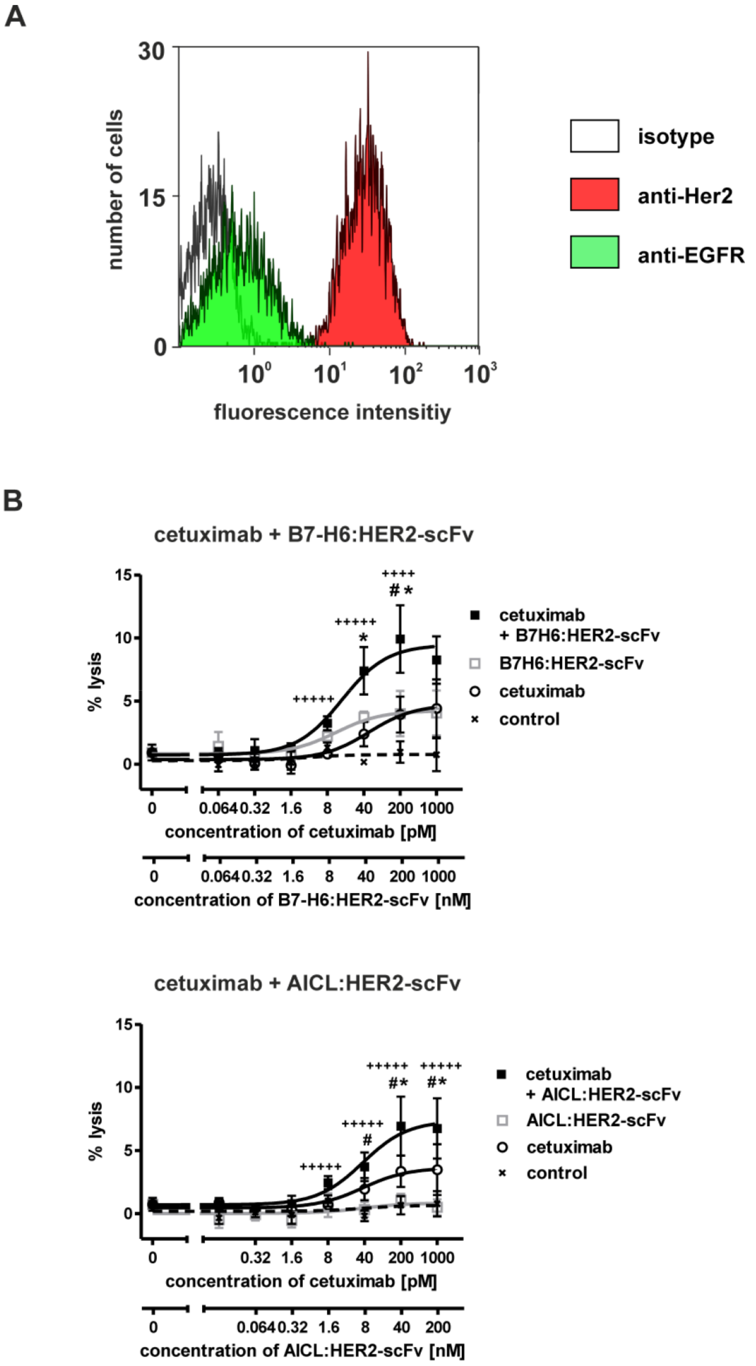

Supplementary Figure S5: Killing of MDA-MB-361 cells by combinations of immunoligands and cetuximab. A. HER2 and EGFR are co-expressed by MDA-MB-361 cells. Surface expression was analyzed with specific antibodies and flow cytometry. **B.** Dose effect curves for pairwise combinations of cetuximab with either B7-H6:HER2-scFv or AICL:HER2-scFv. The effects obtained in combination were compared to the effects achieved by the single molecules at varying concentrations. MNC served as an effector cell source. Rituximab was employed as control. Data points represent mean values ± SEM from at least four independent experiments. Synergistic effects are indicated according to their strength (+++++, CI < 0.1, ++++, CI < 0.3; +++, CI < 0.7). Statistically significant differences between combinations and immunoligands (#*P* < 0.05) or between combinations and the antibody (**P* < 0.05) are indicated.
